# Whitening Efficacy and Enamel Protection of 35% Hydrogen Peroxide Solution Containing Alkaline-Substituted Bioactive Glasses

**DOI:** 10.1021/acsomega.5c12119

**Published:** 2026-07-01

**Authors:** Ji Yun Kim, Hae Jeong Kim, Ro Mi Choi, Song-Yi Yang

**Affiliations:** Department of Dental Hygiene, Konyang University College of Medical Science, 158 Gwanjeodong-ro, Seo-gu, Daejeon 35365, Republic of Korea

## Abstract

This study aimed to investigate the whitening efficacy and enamel surface protective effects of 35% hydrogen peroxide (HP) solutions modified with alkali-substituted bioactive glasses (BAGs). Three alkali BAG formulations (K-, Li-, and LiK-based) were added to 35% HP at concentrations of 0.1, 1.0, and 10.0 wt %. The control group contained HP without BAG or distilled water (DW). The pH of each solution was monitored. Discolored bovine enamel specimens were treated thrice with each solution (20 min/session). Enamel surface color change (Δ*E*) was assessed by spectrophotometry, while surface gloss, roughness (Ra), and Vickers microhardness were also measured. Morphological alterations were examined by scanning electron microscopy (SEM). Statistical analysis was performed using one-way analysis of variance with Tukey’s post hoc test (α = 0.05). All BAG-modified groups showed significantly higher pH values than HP alone (*p* < 0.05), with K- and LiK-based formulations producing the greatest pH increases. Except for Li 0.1%, the Δ*E* values of all formulations were statistically comparable to those of HP (*p* > 0.05), indicating no loss of whitening efficacy. BAG addition significantly improved gloss and microhardness and reduced roughness compared with HP alone (*p* < 0.05). At concentrations ≥ 1%, enamel surface properties were restored to levels similar to those of DW (*p* > 0.05). SEM revealed progressively smoother enamel surfaces with increasing BAG concentration. In conclusion, incorporating alkali-substituted BAGs into 35% HP preserved bleaching efficacy while markedly reducing enamel surface damage; concentrations ≥ 1% showed the most consistent protective effects. High-concentration HP bleaching may compromise enamel integrity despite its whitening effectiveness. The addition of alkali-substituted BAGs effectively buffered the bleaching environment and promoted surface protection without impairing whitening efficacy. This approach represents a promising adjunct for safer and more predictable in-office bleaching procedures.

## Graphical abstract

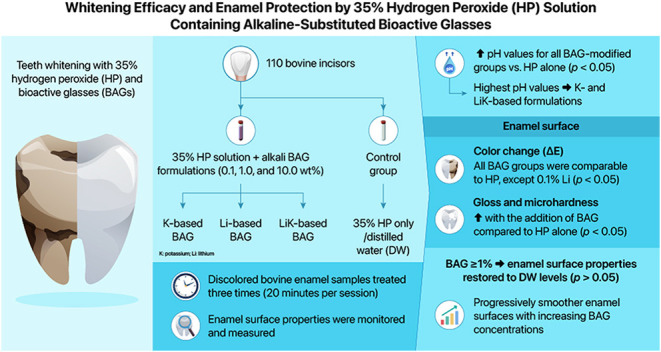

## 1 Introduction

According to the U.S. Food and Drug Administration, tooth whitening refers to the use of chemical or physical methods to remove intrinsic or extrinsic stains from the tooth surface or alter the color of teeth to achieve a brighter appearance.^1^ Currently, hydrogen peroxide (HP) and carbamide peroxide are the most widely used active whitening agents. High-concentration HP (e.g., 35%) is used in in-office bleaching procedures because of its ability to achieve rapid results with fewer applications. These compounds release reactive oxygen species (ROS) upon decomposition, which oxidize chromogenic substances within the enamel and dentin, thereby inducing bleaching.^2,3^

Despite its efficacy, high-concentration HP has been associated with several adverse effects, such as a reduction in enamel microhardness, an increase in surface roughness (Ra), and demineralization, primarily because of its strong oxidative potential and low pH.^4,5^ These physicochemical alterations have also been associated with undesirable clinical outcomes such as increased dentin hypersensitivity and pulpal irritation following bleaching. To address these adverse effects, various remineralizing agents have been incorporated into whitening formulations;^6,7^ however, conflicting results have been reported regarding their efficacy, with some studies indicating limited benefits, particularly under acidic or demineralized conditions.

To overcome these limitations, alternative inorganic additives, such as calcium phosphates, hydroxyapatite, hydrated calcium silicate, and bioactive glass (BAG), have been proposed as candidates for minimizing enamel damage while maintaining whitening efficacy.^8–10^ Among these, BAG has shown the potential to enhance enamel resistance to acid attack by releasing calcium and phosphate ions in aqueous environments and forming a surface layer of hydroxycarbonate apatite (HCA), which is structurally similar to natural enamel.^11^ Since its development by Hench in 1969, BAG has been widely used in bone regeneration and dental applications owing to its excellent biocompatibility and capacity for interfacial bonding with hard tissues.^11,12^ One of the most well-known compositions, 45S5 BAG (containing SiO_2_, Na_2_O, CaO, and P_2_O_5_), exhibits rapid ion release and efficient HCA formation, contributing to both remineralization and neutralization of acidic environments.^13–15^

However, the high content of Na_2_O in 45S5 BAG, while beneficial for lowering the melting temperature and accelerating ion release, increases its susceptibility to crystallization during thermal processing. This tendency compromises the ability of the glass to retain its amorphous structure, which consequently may reduce its bioactivity and limit its processability.^16^ To improve the thermal and structural stability of BAG, recent studies have investigated the partial or complete substitution of Na_2_O with alternative alkali oxides such as K_2_O or Li_2_O. These substitutions have been shown to decrease the crystallization tendency, improve thermal processability, and modulate ion-release kinetics through structural densification of the glass network, reflecting the well-known mixed alkali effect in bioactive glasses.^16–18^ In aqueous environments, such structural modifications may facilitate more controlled and sustained release of Ca^2+^ and PO_4_^3–^ ions, potentially enhancing buffering capacity and enamel surface protection under the acidic conditions associated with high-concentration hydrogen peroxide bleaching. In particular, a mixed composition of lithium and potassium has been suggested to provide a favorable balance among mechanical stability, thermal resistance, and sustained bioactivity. However, the use of alkali-substituted BAGs in conjunction with HP-based tooth-whitening agents has not been adequately investigated. While conventional 45S5 BAG has been reported to reduce enamel demineralization and maintain bleaching efficacy when incorporated into peroxide bleaching systems,^15^ the potential influence of alternative alkali substitutions on enamel protection during high-concentration HP bleaching has not yet been systematically investigated.

Therefore, this study aimed to evaluate the effects of novel alkali-substituted BAGs, in which the Na_2_O component of conventional 45S5 BAG (SiO_2_–Na_2_O–CaO–P_2_O_5_) was replaced by K_2_O, Li_2_O, or a combination of K_2_O and Li_2_O (LiK), when incorporated into a 35% HP solution, with a focus on both whitening performance and preservation of enamel surface properties. The alkali BAG powders (K-, Li-, and LiK-substituted) were incorporated into the 35% HP solution at concentrations of 0.1, 1.0, and 10.0%, and their effects on enamel color change (Δ*E*) and surface properties (surface gloss, roughness, microhardness, and morphology) were evaluated. The null hypotheses tested in this study were as follows: (1) there are no significant differences in whitening efficacy among the experimental groups regardless of BAG composition (K, Li, LiK) or concentration (0.1, 1.0, 10.0%); (2) there are no significant differences in enamel surface properties, including gloss, roughness, microhardness, and microstructure, based on the type or concentration of BAG incorporated into the 35% HP solution.

## 2 Materials and Methods

### 2.1 Preparation of Experimental Alkali BAGs

Experimental alkali BAGs based on the SiO_2_–P_2_O_5_–CaO–Na_2_O–Li_2_O/K_2_O system were synthesized using a conventional melt-quenching method.^17^ To investigate the compositional effects, the Na_2_O component of the base glass composition was completely replaced by K_2_O, Li_2_O, or an equimolar combination of both. Reagent-grade SiO_2_, P_2_O_5_, CaCO_3_, K_2_O, and Li_2_O powders (Sigma-Aldrich, St. Louis, MO, USA) were weighed according to predetermined molar ratios and thoroughly mixed (Table 1). Each 40 g batch was presintered at 1250 °C for 1 h in an alumina crucible using a high-temperature electric furnace (LHT 02/17, Nabertherm, Lilienthal, Germany) to ensure decarbonation and homogenization. The presintered mixtures were melted at 1350 °C for 1 h, followed by rapid quenching to room temperature to obtain amorphous glass frits. The frits were ground into fine powders using planetary ball milling (XQM-4A, TENCAN, Hunan, China) for 5 h at 400 rpm, employing zirconia balls of varying diameters (3, 5, 15, and 20 mm) to enhance the grinding efficiency and achieve a uniform particle size distribution.

**Table 1:** Chemical Compositions of the Experimental Potassium (K)-, Lithium (Li)-, and Potassium–Lithium (LiK)-Based Bioactive Glasses (BAGs) Used in This Study (mol %)^a^

	SiO_2_	P_2_O_5_	CaO	Li_2_O	K_2_O
K BAG	46.1	2.6	26.9		24.4
Li BAG	46.1	2.6	26.9	24.4	
LiK BAG	46.1	2.6	26.9	12.2	12.2

a BAG, bioactive glass.

The amorphous phase of the BAG powders was confirmed by X-ray diffraction (XRD; Ultima IV, Rigaku, Tokyo, Japan) over a 2θ range of 10–60° at a scan rate of 2°/min. Particle size distribution was assessed using a laser diffraction analyzer (Bettersize S2, Bettersize Instruments, Dandong, China), with continuous stirring at 1600 rpm and ultrasonic dispersion at 50 W for 2 min. The micromorphological features and elemental compositions were examined by field-emission scanning electron microscopy (FE-SEM) using a microscope (JSM-7800F, JEOL, Tokyo, Japan) equipped with an energy-dispersive X-ray spectrometer (EDS), operated at 10.0 kV and 15,000× magnification.

### 2.2 Incorporation of Alkali BAGs into HP Solution

Experimental HP solutions containing alkaline BAGs were prepared before each experiment. Three types of BAG powders with different compositions were incorporated into a 35% HP solution (Tokyo Chemical Industry Co., Ltd., Tokyo, Japan) at concentrations of 0.1, 1.0, and 10.0 wt %. Each mixture was vortexed for 10 s by using a vortex mixer (VM1; LABTron, Seoul, Korea) to ensure uniform dispersion. Distilled water (DW) and 35% HP without BAG served as controls and were treated under the same conditions (Table 2).

**Table 2:** Compositions of the Control and Experimental Bleaching Formulations Containing 35% Hydrogen Peroxide (HP), Distilled Water (DW), and Bioactive Glass (BAG) Used in This Study (wt %)^a^

		35% HP (%)	DW (%)	BAG (%)
control	DW	0	100.0	0
	HP	100.0	0	0
K BAG	K 0.1%	99.9	0	0.1
	K 1%	99.0	0	1.0
	K 10%	90.0	0	10.0
Li BAG	Li 0.1%	99.9	0	0.1
	Li 1%	99.0	0	1.0
	Li 10%	90.0	0	10.0
LiK BAG	LiK 0.1%	99.9	0	0.1
	LiK 1%	99.0	0	1.0
	LiK 10%	90.0	0	10.0

a BAG, bioactive glass; HP, hydrogen peroxide; and DW, distilled water.

### 2.3 Evaluation of pH Change

The pH changes in the 35% HP solutions containing the three types of compositionally modified BAG powders were evaluated using a pH meter (Orion Star A211, Thermo Fisher Scientific, Singapore) calibrated with standard buffer solutions (pH 4.01, 7.00, and 10.01; Orion Buffer, Thermo Fisher Scientific) according to the manufacturer’s instructions. Preweighed amounts of BAG powder were incorporated into 35% HP solutions according to the experimental design, and all mixtures, including the control groups, were vortexed for 10 s to ensure uniform dispersion. The pH electrode was then immersed in each solution, and the pH values were recorded at 1 min intervals for a total duration of 20 min to monitor temporal changes.

### 2.4 Preparation of Bovine Enamel Specimens

Overall, 110 bovine incisors were obtained from a commercial biological resource supplier (TSS; Incheon, Korea). Teeth with visible enamel defects, such as caries, cracks, or erosion, were excluded after visual inspection. The crowns were separated from the roots using a diamond-coated disk mounted on a micromotor (Marathon-4, Saeyang Microtech, Daegu, Korea), and each crown was embedded in self-curing acrylic resin (Polycoat EC-304, Aekyung Chemical, Chungnam, Korea) using cylindrical Teflon molds (3.0 cm diameter, 1.0 cm thickness). Resin polymerization was conducted at 25 ± 1 °C for 24 h, after which the specimens were removed from the molds. To obtain a flat and uniform enamel surface, the specimens were sequentially polished using silicon carbide abrasive papers (#1200, #1500, #2000 grit; Deerfos, Incheon, Korea) in a water-cooled polishing unit (Ecomet30, Buehler, Lake Bluff, IL, USA). The polished specimens were randomly allocated to experimental groups (*n* = 10 per group).

For enamel staining, each specimen was individually immersed in a tea solution prepared by steeping a 2 g tea bag (Twinings Earl Gray, UK) in 200 mL of boiling DW for 4 min. Specimens were placed in separate wells of a 6-well plate (SPL Life Sciences, Pocheon, Korea) and incubated in freshly prepared tea solution, which was replaced every 24 h for 5 days at 37 ± 1 °C and 100% relative humidity. After staining, the specimens were rinsed with DW for 10 s and dried in compressed air for 10 s.

### 2.5 Bleaching Procedure

For bleaching, predetermined amounts of BAG powder were incorporated into a 35% HP solution and vortexed for 10 s to ensure uniform mixing. A 120-μL aliquot of the freshly prepared experimental solution was then applied to the tea-stained enamel surface of each specimen using a micropipette, ensuring full coverage. The solution was left on the enamel for 20 min, after which the specimens were rinsed with DW and dried in compressed air for 10 s. This procedure was repeated three times under identical conditions, with fresh solutions prepared immediately before each application. After the final bleaching cycle, all the specimens were immersed in DW, ultrasonically cleaned for 5 min, and air-dried for 10 s.

### 2.6 Color Measurement of Enamel Surfaces

Color measurements were performed using a spectrophotometer (CM-5, Konica Minolta, Osaka, Japan) with a 3.0 mm aperture. Each specimen was securely positioned to ensure consistent contact with the measuring port. Three random points were measured per specimen, and the average L*, a*, and b* values in the CIE Lab* color space were recorded. In this system, L* indicates the lightness, a* represents the red–green axis, and b* indicates the yellow–blue axis. Color values were obtained before and after bleaching, and the overall color change (ΔE*) was calculated using the following formula: where ΔE* = [(ΔL*)^2^ + (Δa*)^2^ + (Δb*)^2^]^1/2^

### 2.7 Gloss Measurement of Enamel Surfaces

The gloss of the bleached enamel surfaces was measured in gloss units (GU) by using a gloss meter (Novo-Curve, Rhopoint Instruments, East Sussex, UK). The device was calibrated before measurement using standard calibration tiles with smooth and rough surfaces, according to the manufacturer’s instructions. For each specimen, gloss readings were obtained from three randomly selected regions, and the mean value was recorded as the representative gloss of the specimen.

### 2.8 Roughness Measurement of Enamel Surfaces

The Ra of the bleached enamel specimens was measured using a three-dimensional surface profilometer (Contour GT-X3 Base, Bruker, Germany) in the vertical scanning interferometry mode at 50× magnification. Ra, defined as the arithmetic mean of absolute height deviations from the mean center line, was measured at three randomly selected areas (0.2 × 0.2 mm^2^) per specimen. The mean of the three values was recorded as the representative Ra of each specimen.

### 2.9 Vickers Microhardness Measurement of Enamel Surfaces

Surface microhardness of the bleached enamel specimens was measured using a Vickers hardness tester (MMT-X, Toshima, Akita, Japan). A vertical load of 200 g was applied for 15 s to create indentations, and the diagonal lengths were measured under a microscope at 400× magnification. Three indentations were made at random locations per specimen, and the mean of the three values was recorded as the representative Vickers hardness number (VHN).

### 2.10 SEM Analysis of Enamel Surfaces

The surface micromorphology of the bleached enamel specimens was examined by scanning electron microscopy (SEM; S-3000N, Hitachi, Tokyo, Japan). Before imaging, specimens were coated with a ∼100 nm layer of platinum using an ion sputter coater (E-1010, Hitachi, Tokyo, Japan). SEM images were obtained at magnifications of 2000× and 10,000× to evaluate the surface characteristics of each experimental group.

### 2.11 Statistical Analysis

Data for pH, color change (ΔE*), gloss, Ra, and microhardness were statistically analyzed using one-way analysis of variance (ANOVA), followed by Tukey’s post hoc test for multiple group comparisons. One-way ANOVA was used because the primary objective of the study was to compare the overall performance of each bleaching formulation relative to the clinical reference controls rather than to isolate the independent effects of BAG type and concentration. Analyses were performed using SPSS (version 25.0, IBM, Armonk, NY, USA). Statistical significance was set at *p* < 0.05.

## 3 Results

### 3.1 Powder Characterization of the BAGs

The XRD patterns of the three types of BAGs are shown in Figure 1a. The absence of sharp diffraction peaks across all the samples confirmed the amorphous structure of the synthesized BAGs. Figure 1b shows the particle size distribution of each BAG type, expressed as D10, D50, and D90 values, where D10 represents the particle diameter below which 10% of the total particle volume is found; D50 corresponds to the median particle size; and D90 indicates the diameter below which 90% of the particles are distributed. The K BAG exhibited D10, D50, and D90 values of 1.137, 3.788, and 13.560 μm, respectively. For the Li BAG, the values were 1.202, 5.005, and 18.830 μm, while the LiK BAG showed slightly larger sizes with values of 2.135, 6.839, and 16.510 μm, respectively.

**Figure 1:**
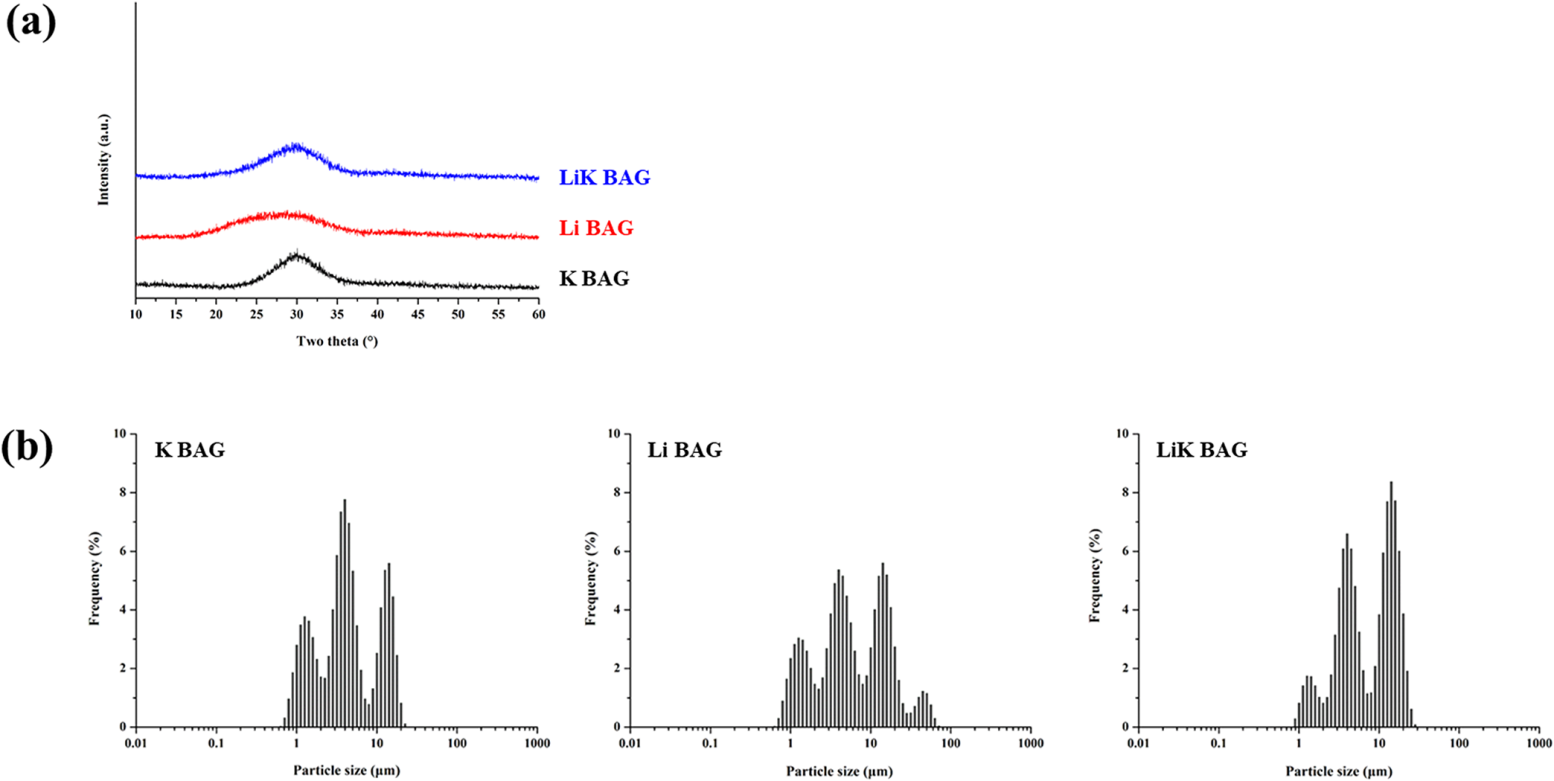
(a) X-ray diffraction (XRD) patterns and (b) particle size distribution profiles of K-, Li-, and LiK-based bioactive glasses (BAGs), illustrating the amorphous structure and particle size characteristics of the synthesized alkaline-substituted BAG formulations.

Figure 2 illustrates the surface morphology and elemental composition of the three BAG types as analyzed by FE-SEM combined with EDS. SEM images acquired at 15,000× magnification revealed that all BAG particles displayed irregular shapes with sharp edges and formed agglomerates with interspersed nanoparticles. EDS analysis confirmed the presence of silicon (Si), calcium (Ca), and phosphorus (P) in all the BAG samples. Potassium (K) was exclusively detected in the K BAG (Figure 2a) and LiK BAG (Figure 2c), while lithium (Li) was not detected in either the Li BAG (Figure 2b) or LiK BAG (Figure 2c). The absence of Li signals may be attributed to the limitations of EDS, which is less sensitive to lighter elements than Beryllium (Be).^19,20^

**Figure 2:**
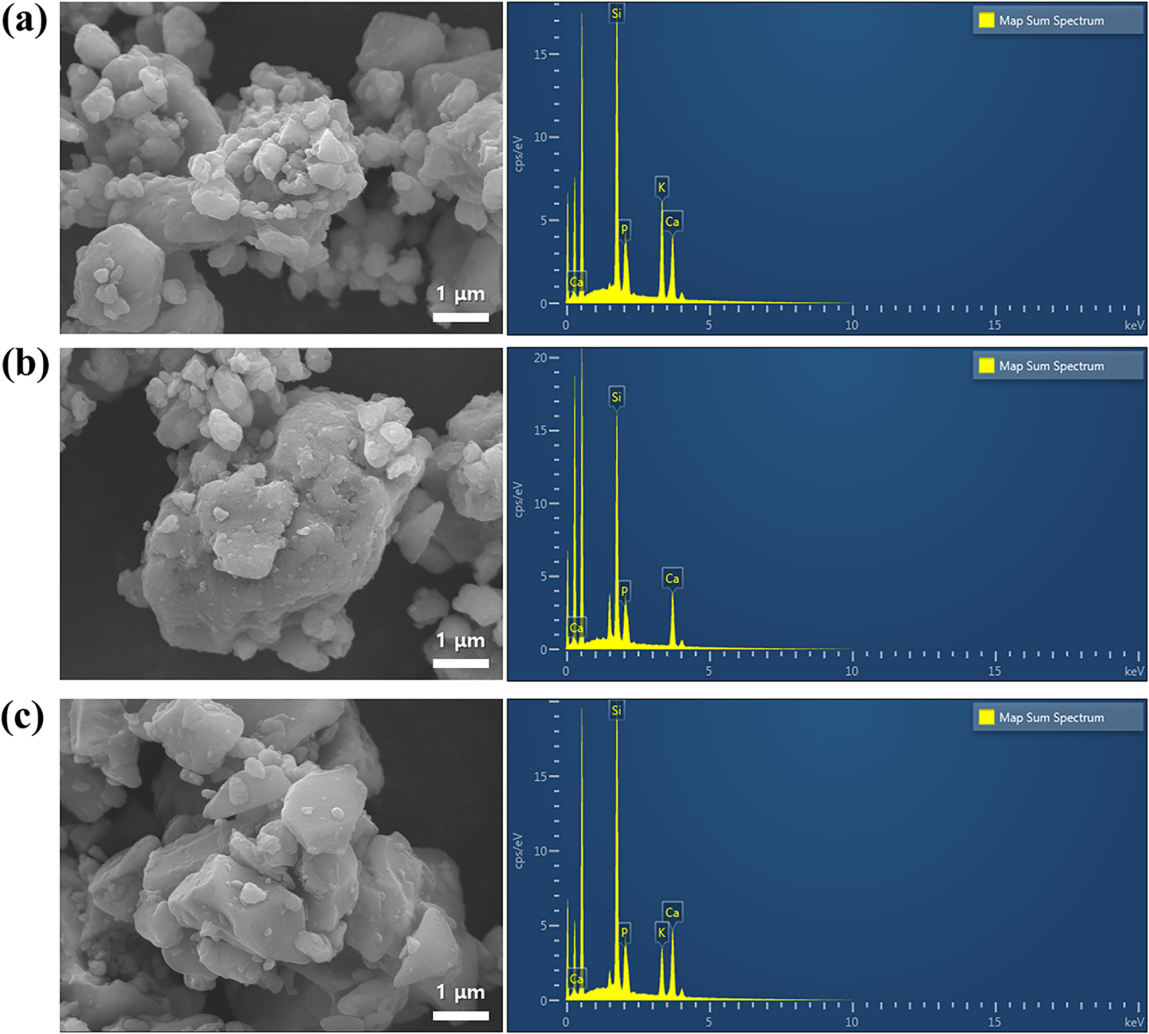
Field-emission scanning electron microscopy (FE-SEM) images and corresponding energy-dispersive X-ray spectroscopy (EDS) spectra of potassium K-, Li-, and LiK-based bioactive glasses (BAGs) powders: (a) K BAG, (b) Li BAG, and (c) LiK BAG. The SEM images illustrate the particle morphology of the synthesized alkaline-substituted BAGs, while the EDS spectra confirm the presence of characteristic constituent elements.

### 3.2 pH Changes in the Control and BAG-Containing Groups

The pH changes following the incorporation of the K, Li, and LiK BAGs into the 35% HP solution are presented in Figure 3. The HP-only control group consistently maintained an acidic pH of approximately 2.5 throughout the 20 min observation period. In contrast, all BAG-incorporated groups exhibited a rapid and significant increase in pH within 1 min of mixing (*p* < 0.05), with initial values of 7.56 ± 0.03 for K 10%, 7.20 ± 0.06 for LiK 10%, and 6.98 ± 0.10 for Li 10%. The elevated pH remained stable for the remainder of the experiment and did not show any statistically significant time-dependent changes (*p* > 0.05). After 20 min, the final pH values showed a concentration-dependent increase for all BAG types (*p* < 0.05). In the K group, pH values rose from 5.14 ± 0.28 (0.1%) to 6.75 ± 0.12 (1%) and 7.58 ± 0.03 (10%). Similarly, the Li group showed values of 4.76 ± 0.16, 6.27 ± 0.12, and 6.92 ± 0.02, while the LiK group recorded 6.02 ± 0.07, 6.65 ± 0.10, and 7.55 ± 0.06 at the corresponding concentrations. At 0.1%, significant differences among the BAG types were observed in the order Li < K < LiK (*p* < 0.05). At 1 and 10%, Li consistently resulted in lower pH values than both the K and LiK BAGs (*p* < 0.05), whereas no significant difference was found between the K and LiK groups (*p* > 0.05). These statistically significant pH increases indicate that the incorporation of alkaline-substituted BAGs effectively neutralizes the acidic HP environment in a concentration-dependent manner, whereas the absence of time-dependent changes suggests rapid stabilization of the buffering effect after initial mixing.

**Figure 3:**
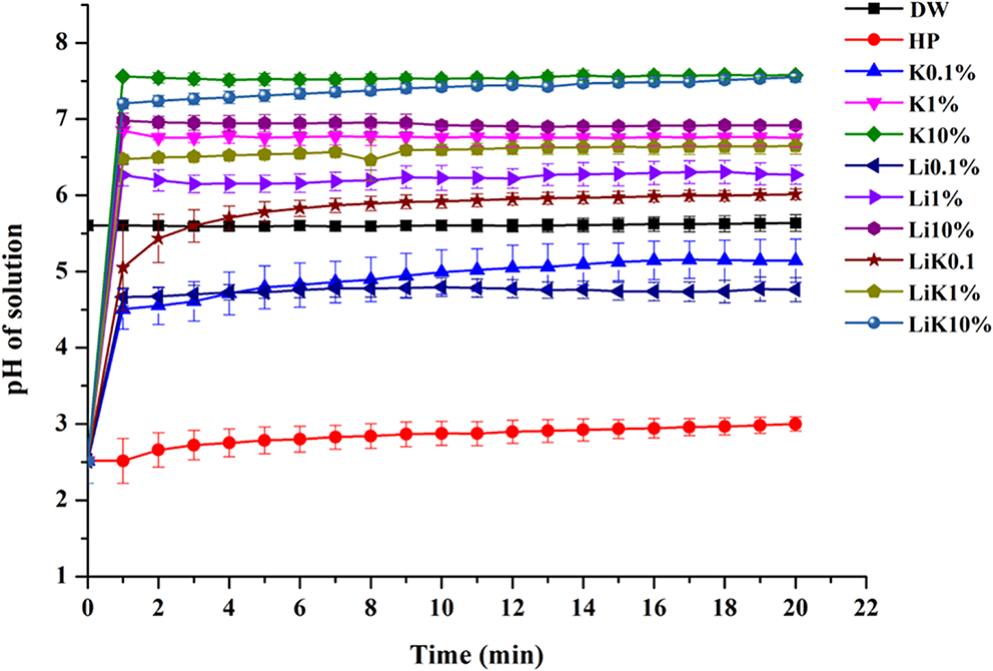
Time-dependent pH changes in 35% hydrogen peroxide (HP) solutions containing various concentrations (0.1, 1.0, and 10.0% w/w) of K-, Li-, and LiK-based bioactive glasses (BAGs), measured over a 20 min period at 1 min intervals. Control groups included HP alone and distilled water (DW). The HP-only control maintained a stable acidic pH of approximately 2.5 throughout the observation period. Each data point represents the mean ± standard deviation (*n* = 6).

### 3.3 Color Measurement

The L*, a*, b*, and Δ*E* values for all experimental groups are summarized in Table 3. “Baseline” refers to the color values after staining (before bleaching), and “Final” refers to the values measured after bleaching. All experimental groups, including the HP-only and BAG-incorporated groups, exhibited significantly higher Δ*E* values than the DW group (*p* < 0.05). Except for the Li 0.1% group, no significant differences in Δ*E* were observed between the BAG-containing and HP-only groups (*p* > 0.05).

**Table 3:** Mean Values and Standard Deviations of Color Coordinates (L*, a*, b*) and Color Difference (Δ*E*) for the Control and Experimental Groups before and after Bleaching Treatment

	L*		a*		b*		
**group**	baseline	final	baseline	final	baseline	final	***ΔE***
**DW**	63.76 ± 3.12	64.48 ± 3.22	3.04 ± 1.16	2.42 ± 0.93	16.79 ± 2.04	16.57 ± 1.85	1.48 ± 0.70^a^
**HP**	63.63 ± 2.56	76.08 ± 3.38	1.96 ± 0.68	–2.11 ± 0.37	13.57 ± 0.74	4.33 ± 1.33	16.32 ± 3.69^b^
**K 0.1%**	63.51 ± 3.40	79.08 ± 1.48	1.89 ± 1.28	–2.24 ± 0.32	15.31 ± 1.68	4.72 ± 1.67	19.49 ± 3.36^bc^
**K 1%**	63.88 ± 1.78	78.37 ± 3.67	1.75 ± 1.24	–2.40 ± 0.15	15.65 ± 1.25	3.21 ± 1.17	19.87 ± 2.71^bc^
**K 10%**	64.68 ± 3.62	77.32 ± 2.33	2.81 ± 1.57	–2.02 ± 0.24	16.60 ± 2.48	5.22 ± 0.80	17.75 ± 3.53^bc^
**Li 0.1%**	63.88 ± 2.50	80.21 ± 4.22	3.63 ± 1.08	–2.14 ± 0.49	18.35 ± 0.80	4.55 ± 1.43	22.37 ± 3.47^c^
**Li 1%**	64.18 ± 2.20	79.05 ± 2.93	3.47 ± 1.05	–2.06 ± 0.30	17.88 ± 1.27	3.00 ± 1.53	21.98 ± 2.05^bc^
**Li 10%**	64.52 ± 2.19	78.57 ± 2.64	2.79 ± 1.08	–2.21 ± 0.22	16.31 ± 1.72	5.50 ± 2.11	18.74 ± 3.38^bc^
**LiK 0.1%**	67.36 ± 2.98	81.20 ± 4.44	1.82 ± 1.01	–1.81 ± 0.46	17.77 ± 1.75	7.47 ± 3.39	18.45 ± 2.40^bc^
**LiK 1%**	64.76 ± 1.63	79.02 ± 2.81	1.94 ± 1.28	–1.81 ± 0.96	18.24 ± 1.86	6.24 ± 1.67	18.41 ± 3.60^bc^
**LiK 10%**	65.88 ± 2.42	80.66 ± 4.06	2.02 ± 0.68	–1.49 ± 0.30	16.29 ± 2.34	6.90 ± 2.79	18.58 ± 2.9^bc^

Within each BAG type, no significant differences in Δ*E* were found among the three concentrations tested. Specifically, in the K BAG group, the Δ*E* values were 19.49 ± 3.36 (0.1%), 19.87 ± 2.71 (1%), and 17.75 ± 3.53 (10%) (*p* > 0.05). For the Li BAG group, the values were 22.37 ± 3.47 (0.1%), 21.98 ± 2.05 (1%), and 18.74 ± 3.38 (10%) (*p* > 0.05). The LiK BAG group showed values of 18.45 ± 2.40 (0.1%), 18.41 ± 3.60 (1%), and 18.58 ± 2.90 (10%) (*p* > 0.05). Likewise, when comparing different BAG types at the same concentration, no statistically significant differences in Δ*E* were observed at any concentration level (*p* > 0.05). These findings indicate that neither the concentration nor the chemical composition of BAGs significantly influenced the degree of color change after bleaching. Statistically, the lack of significant differences in Δ*E* values (*p* > 0.05) demonstrates that the addition of BAGs does not interfere with the bleaching efficacy of 35% HP, thereby preserving its intended whitening performance.

Baseline refers to measurements obtained before the bleaching treatment, and Final refers to measurements obtained after the bleaching treatment. Within the Δ*E* column, identical lowercase letters indicate no statistically significant differences among groups, whereas different lowercase letters denote statistically significant differences (one-way ANOVA followed by Tukey’s post hoc test, *p* < 0.05).

### 3.4 Gloss Measurement

The gloss values of the enamel surfaces after bleaching are shown in Figure 4a. The DW group exhibited the highest gloss (79.14 ± 3.96), followed by experimental groups such as K 10% (75.82 ± 2.86), K 1% (74.92 ± 3.29), Li 10% (72.05 ± 5.54), Li 1% (70.52 ± 4.00), LiK 1% (70.41 ± 4.99), LiK 10% (69.58 ± 1.90), LiK 0.1% (68.03 ± 4.77), K 0.1% (65.58 ± 3.07), and Li 0.1% (64.09 ± 2.78). The HP-only group showed the lowest gloss value (48.71 ± 4.70). All BAG-incorporated groups demonstrated significantly higher gloss values than the HP-only group (*p* < 0.05). Among them, the K 10% and K 1% groups did not differ significantly from the DW group (*p* > 0.05), whereas the remaining experimental groups exhibited significantly lower gloss than the DW group (*p* < 0.05).

**Figure 4:**
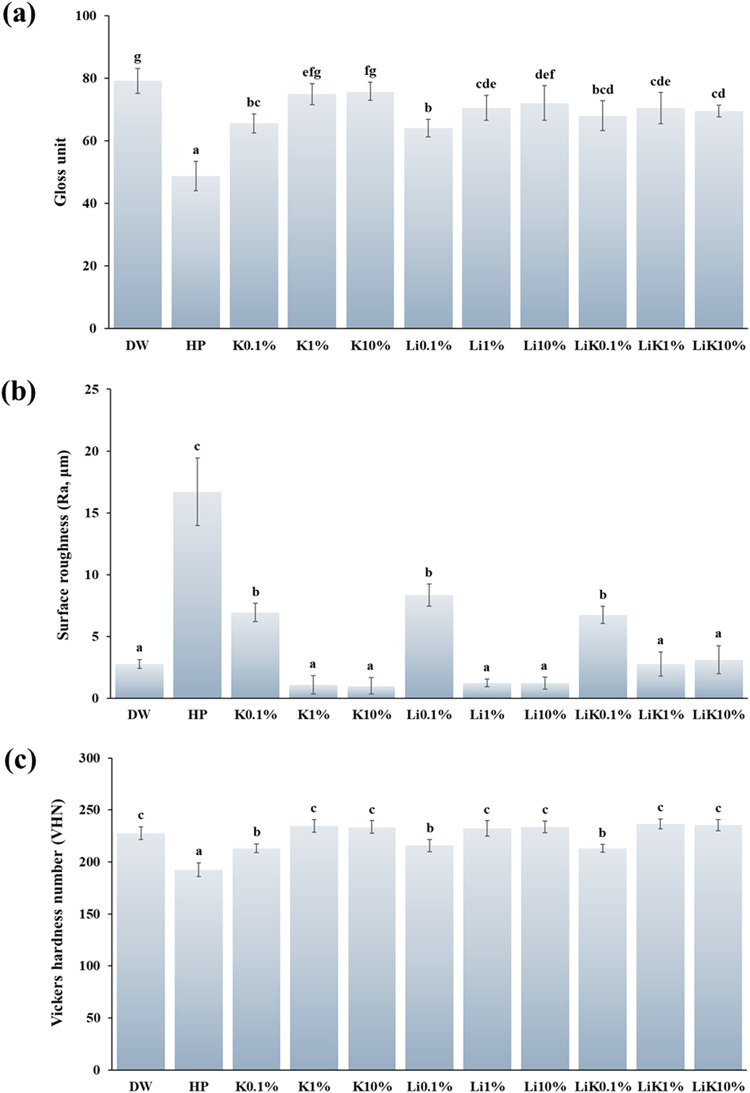
Enamel surface properties measured after bleaching treatment: (a) gloss unit (GU), (b) surface roughness (Ra, μm), and (c) Vickers microhardness number (VHN) in distilled water (DW), hydrogen peroxide (HP)-only, and bioactive glass (BAG)-incorporated experimental groups. Each value represents the mean ± standard deviation of 10 repeated measurements. Statistical analysis was performed using one-way analysis of variance (ANOVA) followed by Tukey’s post hoc test. Identical lowercase letters above the bars denote nonsignificant differences between groups (*p* > 0.05), whereas different letters indicate statistically significant differences (*p* < 0.05).

Regarding the BAG concentration effect, both K and Li groups showed significantly lower gloss at 0.1% than at 1% and 10% (*p* < 0.05), whereas no significant difference was observed between 1 and 10% (*p* > 0.05). In contrast, the LiK group did not exhibit significant differences in gloss across the tested concentrations (*p* > 0.05). When comparing the BAG types at the same concentration, no significant differences were found between the 0.1 and 1% concentrations (*p* > 0.05). However, at 10%, the K BAG group showed significantly higher gloss than the Li K group (*p* < 0.05). The statistically higher gloss values observed in the BAG-incorporated groups compared to the HP-only group (*p* < 0.05) indicate that BAG addition significantly mitigates surface deterioration induced by high-concentration HP, while nonsignificant differences relative to the DW group (*p* > 0.05) suggest effective preservation of near-intact enamel surface characteristics.

### 3.5 Surface Roughness

The Ra of the enamel surfaces after bleaching is shown in Figure 4b. The HP-only group showed the highest roughness (16.71 ± 2.73 μm), which was significantly greater than all BAG-incorporated and DW control groups (*p* < 0.05). Among the groups with 0.1% BAG addition, the Ra values were 8.35 ± 0.90 μm for Li, 6.93 ± 0.74 μm for K, and 6.74 ± 0.71 μm for LiK—significantly lower than the HP group value but higher than those of their respective 1% and 10% groups (*p* < 0.05). At higher BAG concentrations (1 and 10%), Ra values markedly decreased, with values as follows: K 10% (1.01 ± 0.66 μm), K 1% (1.08 ± 0.75 μm), Li 10% (1.22 ± 0.47 μm), Li 1% (1.25 ± 0.32 μm), LiK 1% (2.77 ± 0.97 μm), and LiK 10% (3.11 ± 1.14 μm). These values were comparable to or even lower than those of the DW group (2.78 ± 0.35 μm) and were significantly lower than both the HP-only and 0.1% BAG groups (*p* < 0.05).

Across all BAG compositions, the Ra at 1 and 10% concentrations was significantly lower than that at 0.1% BAG (*p* < 0.05), with no significant difference between the 1 and 10% concentrations (*p* > 0.05). Additionally, no statistically significant differences in Ra were observed between different BAG compositions at the same concentration (*p* > 0.05). These statistically significant reductions in Ra at BAG concentrations of 1% and above (*p* < 0.05) indicate a threshold effect, beyond which further increases in BAG concentration do not yield additional surface-smoothing benefits, as reflected by the absence of significant differences between the 1 and 10% groups (*p* > 0.05).

### 3.6 Surface Microhardness

The VHN of the enamel surfaces following the bleaching treatment is presented in Figure 4c. Among all groups, the highest VHN was observed in the LiK 1% group (236.57 ± 4.79), followed by the LiK 10% (235.36 ± 5.31), K 1% (234.72 ± 6.25), Li 10% (233.82 ± 5.58), K 10% (233.70 ± 6.24), Li 1% (232.48 ± 7.44), and DW groups (227.80 ± 6.09). Lower values were recorded in the 0.1% BAG groups (Li 0.1%: 215.69 ± 5.97, K 0.1%: 213.21 ± 4.23), with the lowest value observed in the HP-only group (192.61 ± 6.67). The VHN of the HP group was significantly lower than that of the other groups (*p* < 0.05).

For all BAG types, specimens treated with 1 and 10% BAG showed no significant difference from the DW group (*p* > 0.05), whereas those treated with 0.1% BAG exhibited significantly lower hardness than those treated with 1 and 10% BAG (*p* < 0.05). Within each BAG type, a similar concentration-dependent trend was observed; the VHN at 0.1% was significantly lower than that at 1 and 10% (*p* < 0.05), whereas no significant difference was found between the VHN at 1 and 10% concentrations (*p* > 0.05). Additionally, no statistically significant differences in the VHN were observed among the three BAG types at any concentration (*p* > 0.05). The statistically significant differences in VHN between the HP-only group and the BAG-containing groups (*p* < 0.05) reflect the substantial protective effect of BAGs against HP-induced enamel softening, whereas the absence of significant differences among BAG types (*p* > 0.05) indicates that the observed hardness preservation is primarily concentration-dependent rather than composition-specific.

### 3.7 Microscopic Analysis of Enamel Surface

Representative SEM images of the enamel surfaces after bleaching are shown in Figure 5. Polishing-induced surface traces were observed in all groups. The HP-only group (Figure 5b) exhibited pronounced surface erosion and a relatively rough morphology compared with the DW control group (Figure 5a), which showed a smooth and uniform surface. In the BAG-treated groups, even at the lowest concentration (0.1%; Figure 5c–e), the enamel surfaces appeared rougher than those in the HP-only group. As the BAG concentration increased, the surfaces became progressively smoother. At 1 and 10% BAG concentrations (Figure 5f–k), the morphology closely resembled that of the DW group, showing a homogeneous and minimally damaged surface.

**Figure 5:**
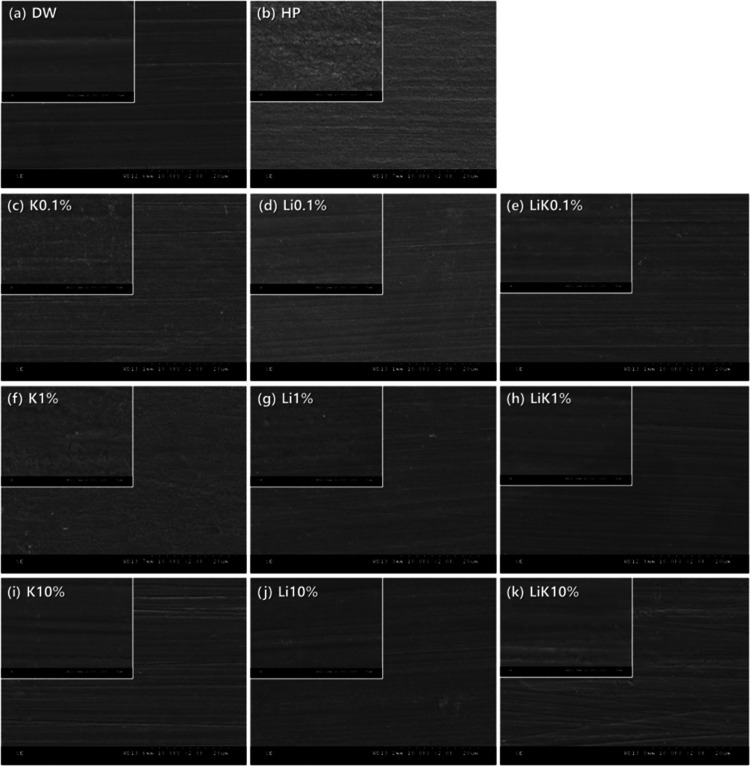
Scanning electron microscopy (SEM) images of enamel surfaces after bleaching treatment in the control and experimental groups, illustrating surface morphological changes and the extent of enamel erosion. Representative images are shown at 2000× magnification (main images) and 10,000× magnification (insets). The groups include distilled water (DW, a), hydrogen peroxide (HP, b), K 0.1% (c), Li 0.1% (d), LiK 0.1% (e), K 1% (f), Li 1% (g), LiK 1% (h), K 10% (i), Li 10% (j), and LiK 10% (k).

When comparing different BAG types at the same concentration, slight differences in the surface texture were observed at 0.1 and 1%, depending on the BAG composition. However, at 10%, all of the BAG types yielded similar smooth enamel surfaces.

## 4 Discussion

Professional tooth bleaching with 35% HP effectively whitens discolored teeth by decomposing or neutralizing the intrinsic organic pigments via powerful oxidation processes.^21^ Clinically, such high-concentration hydrogen peroxide formulations are commonly used in in-office bleaching procedures, where rapid and pronounced whitening effects are required. However, increased HP concentrations result in elevated formation of ROS, and the decomposition byproducts of HP consequently create a highly acidic environment.^22^ Under these acidic conditions, hydroxyapatite—the primary inorganic component of the enamel—begins to dissolve, causing microstructural demineralization and weakening of the chemical bonds between organic and inorganic materials through ROS activity.^23,24^ In particular, repeated bleaching procedures under such conditions can significantly accelerate structural damage and decrease the microhardness of the enamel, leading to increased tooth sensitivity and compromised long-term structural integrity.^25^ Therefore, the development of functional additives that can buffer or neutralize the acidic environment associated with HP-based bleaching agents is crucial.

Previous studies have reported strategies for preventing enamel damage by incorporating hydroxyapatite^26^ or 45S5 BAG^27^ during bleaching. However, these studies primarily utilized animal-derived enamel samples and performed limited evaluations (color change and microhardness), resulting in an insufficient clinical generalizability. Additionally, 45S5 BAG has a high Na_2_O content, leading to rapid dissolution rates and compromised thermal and chemical stabilities.^16,28^ To overcome these limitations, the present study developed novel alkaline-substituted BAG formulations based on K, Li, and LiK to improve their thermal stability and regulate their ion-release characteristics. These BAG variants were incorporated into a 35% HP bleaching solution, and comprehensive analyses of the whitening efficacy and enamel surface protection were conducted by using multiple physical and optical parameters.

Previous research has demonstrated that 45S5 BAG-containing Na_2_O effectively neutralizes acidic environments, thus inhibiting enamel demineralization.^29^ An alkaline environment not only prevents demineralization but also promotes remineralization, restoring enamel strength. It has been reported that fluoride incorporated under conditions of pH 6.8 resulted in greater remineralization than at pH ≤ 5.5.^30^ In the current study, except for the low-concentration (0.1%) K and Li BAG groups, most BAG-containing groups exhibited a final pH above 5.5, which was significantly higher than that of the HP-only group (*p* < 0.05). The observed pH elevation is likely associated with the release of alkaline ions (K^+^, Li^+^) from BAG, which can contribute to buffering the acidic HP environment through proton consumption. Furthermore, Ca^2+^ and PO_4_^3–^ ions released from BAG may contribute to surface mineral deposition processes that are commonly associated with HCA layer formation on the enamel surface, subsequently filling microdefects, preventing further demineralization, and contributing to structural stability and microhardness maintenance.^31^

Additionally, previous studies involving commercial dental products containing BAG have reported positive outcomes.^32,33^ A previous study showed that adding 10 wt % F-18 BAG to a 35% HP gel resulted in hydroxyapatite deposition, localized pH elevation, antimicrobial effects, and significant inhibition of enamel microhardness loss, while preserving the bleaching efficacy.^32^ Another study demonstrated that BAG-based toothpaste, applied either before or after bleaching with 35% HP, effectively prevented the reduction in enamel microhardness without compromising bleaching efficacy. These observations are consistent with the results of the current investigation and provide additional evidence that BAG can function as a protective agent against enamel surface damage induced by high-concentration HP bleaching procedures.^33^ These previous investigations support the potential clinical relevance and translational promise of the current study, wherein novel K, Li, and LiK BAG formulations replaced the conventional Na-based BAG. Previous studies have demonstrated that conventional Na-based 45S5 BAG incorporated into HP bleaching systems can effectively reduce enamel demineralization while maintaining whitening efficacy.^15,27^ In the present study, the whitening outcomes (Δ*E* = 17.75–21.98) and the recovery of enamel surface properties such as roughness and microhardness at BAG concentrations ≥1% were consistent with the protective trends reported in those studies. Although the present investigation focused on alkali-substituted BAG formulations rather than conventional 45S5 BAG, these comparable outcomes suggest that the modified BAG compositions may provide similar enamel-protective functionality under high-concentration hydrogen peroxide conditions. By systematically evaluating color change (Δ*E*), pH variations, surface gloss, roughness, microhardness, and SEM microstructural changes, this study comprehensively assessed the impact of BAG composition and concentration on enamel protection and whitening outcomes.

Sulieman et al. introduced a reproducible *in vitro* staining model to quantitatively evaluate bleaching efficacy,^34^ and a modified version of this methodology was applied in the present study. The hydrogen peroxide used in this study was prepared on the basis of commercially available products, reflecting key aspects of professional bleaching formulations under controlled experimental conditions. Distilled water was used instead of artificial saliva to minimize external remineralization effects, thereby enabling a controlled assessment of the functional effects of BAG incorporation on the bleaching environment.^35,36^ The bovine enamel specimens utilized in the current study, which have been extensively validated as substitutes for human enamel because of their structural and compositional similarities, provided a scientifically robust basis for comparative evaluation.^37^ While this standardized *in vitro* approach does not fully replicate the complexity of the clinical oral environment, it allows for a reliable and reproducible method for a controlled comparative evaluation of whitening efficacy among different bleaching formulations.

The first null hypothesis, “Addition of K, Li, and LiK-based BAG to 35% HP would not result in significant differences in whitening efficacy (Δ*E*)” was accepted. All BAG-containing groups showed color changes (Δ*E*) statistically comparable to the HP-only group, irrespective of the BAG concentration (0.1, 1.0, and 10.0%) (*p* > 0.05). Clinical bleaching efficacy assessments typically employ shade guides or instrumental colorimetric analysis.^38^ In the present study, L* values significantly increased, and b* values decreased after bleaching, except in the DW control group, indicating enhanced brightness and reduced yellowness. In contrast, the DW control group exhibited negligible Δ*E* values.^2,39^ Thus, BAG incorporation does not interfere with HP bleaching mechanisms involving radical generation and oxidative cleavage of organic pigments.^2^ From a clinical standpoint, this suggests that the addition of alkaline-substituted BAGs does not compromise the whitening efficacy expected from in-office bleaching treatments using high-concentration HP.

Tooth gloss—an optical characteristic determined by the degree of light reflected from the enamel surface—is a crucial element in assessing esthetic outcomes following tooth bleaching. Although gloss can be visually perceived, objective quantitative analysis using specialized instrumentation is essential to minimize interobserver variability.^40,41^ Gloss is a reliable indicator for detecting subtle enamel changes induced by bleaching treatments.^42,43^ In the present study, gloss changes in enamel surfaces treated with 35% HP solutions containing various concentrations of K, Li, and LiK BAG were measured using a gloss meter. The DW group showed negligible gloss alterations from before to after treatment, whereas both the HP-only and BAG-containing groups exhibited an overall reduction in gloss. However, enamel treated with BAG-containing formulations maintained significantly higher gloss values compared to the HP-only group (*p* < 0.05). At some compositions and concentrations, the gloss levels were statistically similar to those in the DW group (*p* > 0.05). These findings suggest that incorporating BAG into HP effectively mitigates enamel gloss reduction, likely through acid-buffering effects and the promotion of surface mineralization. Clinically, the preservation of enamel gloss and surface integrity is closely associated with improved esthetic outcomes and reduced risk of surface degradation following repeated in-office bleaching procedures. In a related study, Yalcin et al. reported a significant gloss reduction even with lower HP concentrations (6.5%), highlighting gloss deterioration as a common consequence of tooth bleaching, regardless of the HP concentration.^42^ Compared to these studies, our finding that high-concentration HP bleaching agents containing BAG can effectively preserve enamel gloss has significant implications for clinical aesthetics and structural stability.

Enamel Ra and microhardness are primary indicators for quantitatively assessing structural damage following tooth bleaching procedures. During the bleaching process, oxidative reactions affect both the organic proteins and inorganic mineral structures of the enamel, potentially increasing the Ra and subsequently enhancing plaque adhesion, staining susceptibility, and the loss of gloss. Similarly, microhardness is indicative of the enamel’s durability and resistance. Thus, reduced hardness is directly correlated to mineral loss and structural compromise, posing the risk of enamel weakening through repeated bleaching procedures. Consequently, gloss, roughness, and microhardness constitute essential combined evaluation parameters for assessing enamel structural and functional changes after bleaching, as previously highlighted in the context of dental surface analysis.^41^ In the present study, the Ra values were measured using a noncontact surface profilometer, whereas microhardness was analyzed using a Vickers hardness tester. The results demonstrated that the HP-only group exhibited the highest Ra value and the lowest VHN, indicating structural damage and demineralization caused by the high-concentration HP treatment. In contrast, the enamel treated with BAG-containing HP solutions showed a concentration-dependent reduction in Ra and an increase in the VHN (*p* < 0.05), which were statistically indistinguishable from those in the DW group at concentrations of 1.0% and above (*p* > 0.05). These results highlight the role of BAG in mitigating enamel surface deterioration and maintaining the mechanical integrity after bleaching. Similar trends have been reported in previous studies; for example, de Arruda et al. noted significantly decreased microhardness following 35% HP application,^44^ and Ghafir et al. observed increased Ra under similar bleaching conditions.^45^ Although our findings align with those of previous studies, adding BAG provides further evidence of its efficacy in effectively reducing the physical damage induced by HP bleaching.

SEM revealed pronounced enamel erosion and rough and uneven surface structures in the HP-only group after bleaching. These observations are consistent with those reported by Deng et al.,^27^ who documented significant surface erosion and tissue loss after 35% HP bleaching, and those of de Arruda et al.,^44^ who observed porous enamel structure formation under similar conditions. These results collectively indicate that high-concentration bleaching agents induce microscopic erosion, compromising enamel surface integrity and smoothness. However, in the present study, the BAG-containing groups demonstrated markedly reduced erosion compared to the HP-only group. Particularly, at concentrations of 1.0% and higher, SEM images revealed smoother and more uniform surfaces, comparable to those observed in the DW control group. These results suggest that incorporating BAG into HP is associated with reduced enamel demineralization and improved surface integrity under high-concentration bleaching conditions. Thus, SEM analysis provides valuable visual confirmation of the physical protective effects of BAG addition, supplementing qualitative assessments and enabling the comparative evaluation of enamel responses to various bleaching formulations and concentrations. The D90 values of the synthesized BAGs ranged from 13.56 to 18.83 μm. Although the potential for surface abrasion during repeated clinical application cannot be entirely excluded, the Ra values in all BAG-containing groups were significantly lower than those in the HP-only group and were statistically comparable to the DW control at concentrations ≥ 1%. These findings provide indirect evidence that BAG incorporation did not induce additional enamel abrasion under the present experimental conditions. Nevertheless, the physicochemical behavior of BAG particulates in clinical oral environments warrants further investigation.^46^

Based on these findings, the second null hypothesis, that increasing the concentration of K, Li, and LiK BAG added to 35% HP would not yield significant differences in enamel surface characteristics (gloss, roughness, microhardness, and microstructure), was rejected. Indeed, increasing the BAG concentration significantly enhanced enamel surface properties, providing consistent evidence of dose-dependent protective effects. Specifically, at concentrations exceeding 1.0%, enamel surface characteristics remained stable compared to the HP-only group, with the 10.0% K BAG group notably demonstrating both a high final pH and equivalent bleaching efficacy (Δ*E*) compared to the HP-only group. This indicates that alkaline BAG additives simultaneously achieve enamel protection and maintain bleaching effectiveness, emphasizing the importance of optimizing the BAG composition and concentration for effective and safe bleaching formulations. Overall, the present findings demonstrate that alkaline-substituted BAGs can be incorporated into high-concentration hydrogen peroxide formulations used for in-office bleaching, providing enamel-protective benefits while maintaining clinically relevant whitening efficacy. These observed enamel surface responses highlight the potential of optimized BAG-containing bleaching formulations as a strategy to mitigate enamel damage associated with professional whitening procedures. Such approaches align with broader research trends in dental materials, where functional inorganic additives and nanoparticle-based fillers are increasingly explored to enhance material performance and bioactivity.^47^

In line with previous reports showing that BAG-modified bleaching agents or adjunctive BAG-containing products can preserve enamel microhardness and surface integrity without compromising whitening efficacy under high-concentration peroxide conditions,^32,33^ the present findings further support the clinical promise of mineral-based additives in professional bleaching systems. Notably, this study extends the current evidence base by systematically comparing alkali-substituted BAG compositions (K-, Li-, and LiK-based) and concentrations under identical 35% HP conditions, providing additional insights into composition- and dose-dependent protective effects. Compared with prior work largely focused on conventional Na-based BAGs or single formulations, the present results suggest that alternative alkali substitutions may offer a useful strategy for optimizing enamel protection in high-concentration, in-office bleaching applications.

Although direct measurement of HP decomposition kinetics was beyond the scope of this study, the rapid pH elevation induced by BAG (up to ∼ 7.5) may accelerate ROS generation via preferential formation of the hydroperoxide anion (HOO^–^) under alkaline conditions.^2^ Despite this, equivalent Δ*E* values across all BAG groups (*p* > 0.05) indicate that sufficient oxidative capacity was maintained throughout treatment, consistent with findings reported for 45S5 BAG under comparable conditions.^15^ The effect of BAG-induced pH changes on long-term bleaching gel stability warrants direct investigation in future studies. Instead, the focus was placed on evaluating the functional performance of BAG-containing bleaching formulations under clinically relevant high-concentration HP conditions, based on observed changes in pH modulation and enamel surface responses. Despite these favorable outcomes, certain limitations must be acknowledged when considering the direct clinical application of these findings. This study employed an *in vitro* experimental design primarily intended to evaluate the functional effects of material incorporation under controlled conditions without interference from complex *in vivo* factors, necessitating a cautious interpretation of the clinical generalizability. Specifically, the use of bovine rather than human enamel specimens limits direct extrapolation, as bovine enamel differs from human enamel in prism arrangement and microstructural organization, which may influence its response to bleaching agents and subsequent mineral deposition or surface alterations. Moreover, the long-term cumulative effects of repeated bleaching treatments, sustained retention of BAG particles, and biocompatibility assessments were beyond the scope of this investigation. The absence of a conventional 45S5 BAG control group is a notable limitation, as it precludes definitive attribution of the observed effects to alkali substitution specifically, rather than to the inherent bioactivity of BAG in general. Future studies incorporating 45S5 BAG as an active comparator under identical conditions are warranted to substantiate the compositional advantage of alkali substitution. The potential influence of the BAG-deposited mineral layer on subsequent composite resin bonding to bleached enamel also requires further investigation. In addition, the use of DW instead of artificial saliva limits clinical extrapolation, as salivary constituents in the oral environment would continuously challenge the stability of newly formed HCA layers on enamel surfaces.^48^ Consequently, further studies should incorporate more sophisticated simulations of the oral environment to thoroughly evaluate the long-term cumulative interactions between bleaching agents and BAG, thereby providing a more comprehensive basis for clinical translation.

## 5 Conclusions

This study quantitatively evaluated the effects of incorporating K, Li, and LiK alkaline BAGs (0.1%, 1.0%, and 10.0%) into 35% HP bleaching agents on tooth-whitening efficacy and enamel surface characteristics. The results indicated that BAG addition maintained equivalent bleaching efficacy (Δ*E*) compared to HP-only treatment, while significantly buffering the acidic environment (through increased pH), preserving enamel gloss, reducing Ra, maintaining microhardness, and minimizing surface erosion. Concentrations of 1.0% or higher consistently provided superior enamel stability, and SEM analysis confirmed minimal structural damage. These findings support the practical potential of alkaline BAG incorporation to simultaneously achieve effective tooth whitening and protect enamel structural integrity. Therefore, K-, Li-, and LiK-based BAGs represent promising functional additives for the development of future clinical bleaching formulations aimed at balancing aesthetic outcomes with enamel preservation.
